# 3D Reconstruction of the Intracortical Volume Around a Hybrid Microelectrode Array

**DOI:** 10.3389/fnins.2019.00393

**Published:** 2019-04-24

**Authors:** Aparna Nambiar, Nicholas F. Nolta, Martin Han

**Affiliations:** Department of Biomedical Engineering, University of Connecticut, Storrs, CT, United States

**Keywords:** serial section, image registration, 3D reconstruction, intracortical microelectrode, histology, foreign body response, confocal microscopy, dewarping

## Abstract

Extensive research using penetrating electrodes implanted in the central and peripheral nervous systems has been performed for many decades with significant advances made in recent years. While penetrating devices provide proximity to individual neurons *in vivo*, they suffer from declining performance over the course of months and often fail within a year. 2D histology studies using serial tissue sections have been extremely insightful in identifying and quantifying factors such as astroglial scar formation and neuronal death around the implant sites that may be contributing to failures. However, 2D histology has limitations in providing a holistic picture of the problems occurring at the electrode-tissue interface and struggles to analyze tissue below the electrode tips where the electrode tracks are no longer visible. In this study, we present 3D reconstruction of serial sections to overcome the limitations of 2D histological analysis. We used a cohort of software: XuvStitch, AutoAligner, and Imaris coupled with custom MATLAB programming to correct warping effects. Once the 3D image volume was reconstructed, we were able to use Imaris to quantify neuronal densities around the electrode tips of a hybrid microelectrode array incorporating Blackrock, Microprobes, and NeuroNexus electrodes in the same implant. This paper presents proof-of-concept and detailed methodological description of a technique which can be used to quantify neuronal densities in future studies of implanted electrodes.

## Introduction

Numerous studies have implanted electrodes chronically into the brain to stimulate neurons and record neural activity (Wessberg et al., 2000; Santhanam et al., 2006; Collinger et al., 2013; Downey et al., 2016; McCreery et al., 2018). These studies have produced basic neuroscience discoveries and demonstrated proof-of-concept for brain-machine interfaces such as those restoring upper limb control (Collinger et al., 2013; Downey et al., 2016). However, the electrodes that interface with brain tissue tend to decline in performance over the course of months and in most cases stop recording any single unit action potentials within a year (Liu et al., 1999; Barrese et al., 2013).

Therefore, many investigators have sought to identify the failure modes of microelectrodes in the brain. Breakage of connectors and delamination of insulating materials are significant failure modes, but many failures occur despite a seemingly functional implant, indicating that problems exist on the biological side of the interface as well (Prasad et al., 2012, 2014; Barrese et al., 2013; Nolta et al., 2015; Black et al., 2018; Cody et al., 2018). Several mechanisms have been proposed including: glial encapsulation increasing the distance and impedance between neurons and electrodes (Edell et al., 1992; Liu et al., 1999; Turner et al., 1999); encapsulation, scarring, tissue loss, or tethering forces causing the electrode to move away from its intended position or to tilt within tissue (Liu et al., 1999; Barrese et al., 2013; Nolta et al., 2015; Cody et al., 2018); and death, degeneration, or rewiring of neurons causing them to become silent (Biran et al., 2005; McCreery et al., 2016; Eles et al., 2018).

The primary tool for testing these hypotheses has been histological analysis of tissue near the electrodes. Typically, tissue is sectioned, immunohistochemically-labeled, and analyzed quantitatively as a series of 2D images (Shain et al., 2003; Biran et al., 2005; McConnell et al., 2009; Kozai et al., 2012; Potter et al., 2012). Even when confocal microscopes are used, the 3D images of individual sections are projected into 2D images before analysis. 2D histological approaches have had some success correlating histological features with chronic performance of individual electrodes on large, multi-electrode devices (Freire et al., 2011; Prasad et al., 2012, 2014; Kozai et al., 2014; Nolta et al., 2015; McCreery et al., 2016); however, in these studies, it was not always possible to determine why a particular electrode failed, and teasing apart multiple confounding failure modes proved challenging.

2D histological analysis has significant limitations. The “recording zone” in which neural action potentials can be detected extends approximately 100 μm from the electrode recording sites (Henze et al., 2000), so in 2D histology, this is taken as a 100-μm-radius circle in a tissue section at a depth corresponding to the recording site. However, the recording zone is actually a 3D sphere, including tissue 100 μm below the recording site in sagittal or coronal view. This means the tissue below the recording site is being ignored. For tip-based microelectrodes, the tissue below is of particular interest since this tissue has been less disrupted by the electrode. This cannot be rectified by simply looking at more sections, because in 2D histology, the location of the recording site is unknown once the electrode track is no longer visible. Hence, some healthy neurons in the tissue below the tip contributing to the recordings may go uncounted, which could lead to inaccurate correlation of neuronal density with recording data. This aspect also has ramifications in stimulation applications because virtually all data related to the Shannon-McCreery curve for safe limits of charge injection were collected based on 2D histology (Shannon, 1992).

Another limitation of 2D histology is the difficulty of visualizing and understanding the large scale, depth-dependent features of the biological response, such as the location or tilt of the device within cortical layers, when looking at a series of 2D images. These changes in location or tilt can be very significant due to the forces exerted by connective tissues and stiff tethering cables (Karumbaiah et al., 2013; Nolta et al., 2015). A holistic, 3D view of these problems could provide new insights.

A few recent studies have conducted 3D analysis of tissue near electrodes. One group cleared, stained, and imaged thick sections with the electrode in place, although the imaging depth was limited to about 300 μm (Woolley et al., 2011, 2013; Lee et al., 2018). 3D regions of tissue loss were reconstructed at low resolution from 2D serial sections (Nolta et al., 2015). Micro-CT has been employed to localize electrodes before sectioning (Cody et al., 2018). Finally, 3D two-photon imaging was used to study the chronic biological response *in vivo*, although this required implanting the device at an oblique angle and installing a cranial window (Kozai et al., 2016; Eles et al., 2018).

An alternative approach would be to use software to reconstruct a 3D dataset from confocal images of every section. This would enable 3D analysis of the full implant area without having to alter established histological procedures, and also allow analysis of already-scanned tissues. The main challenge is in aligning consecutive sections. Fully-automated alignment is possible with advanced software and adequate fiducials (Arganda-Carreras et al., 2010) or if there are fine details that can be correlated between sections (Mathiisen et al., 2010). Unfortunately, in our case, our available fiducials, such as electrode tracks, blood vessels, and brightly-staining regions of tissue, were not recognizable by a professional automatic alignment software package Voloom (Bitplane AG, Zurich, Switzerland). It was also clear that minor blade losses during sectioning and significant deformation of individual sections during processing precluded the use of alternative programs operating on the correlation of fine details between sections. Our strategy instead was to use the electrode tracks as fiducials and perform alignment manually. Our device had electrode sites at the tips of 1-mm-long electrodes, so we were able to reconstruct the tissue above and below the tips of all the 1-mm electrodes using the 2-mm electrodes as fiducials. Our technique was similar to that of a group performing midbrain mapping studies (Markovitz et al., 2012) but different in that it used 3D confocal images, achieved alignment accuracy much better than 100 μm, and did not require adding artificial fiducials. In addition, we developed and employed a simple dewarping algorithm to help reduce the effects of tissue deformation. Finally, we were able to quantify the density of neuronal nuclei in 3D regions around recording sites, which to our knowledge has not yet been accomplished in our field. This work describes our technique in detail and demonstrates its potential to improve the understanding of biological failure modes of chronically implanted electrodes.

## Materials and Methods

### Devices, Surgery, Immunohistochemistry, and Image Acquisition

**FIGURE 1:**
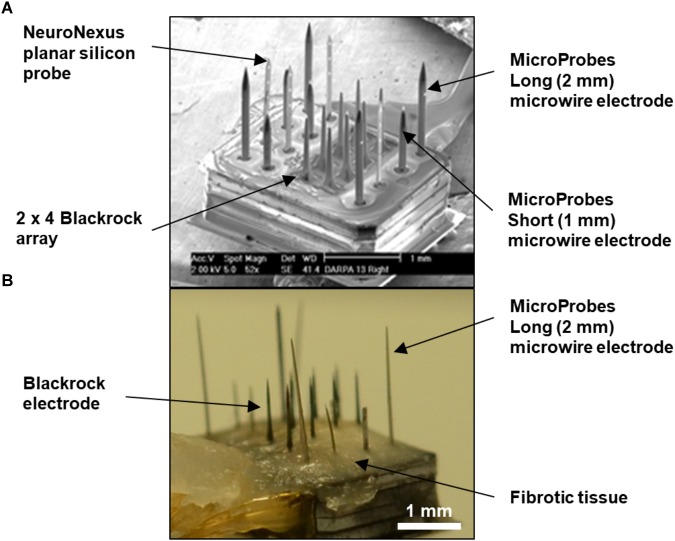
**(A)** SEM of fully-assembled hybrid array before implantation showing four types of commercial microelectrodes assembled in a mixed fashion. The overall footprint of the array is approximately 1.7 mm × 2.2 mm. **(B)** Photograph of the explanted hybrid array from the animal used in this study. The base of the array was covered with fibrotic tissue, but the majority of the length of the electrodes appeared free of tissue. Also, the long microwire and NeuroNexus probes were tilted at an angle.

Raw images were from an unpublished study by our group using “hybrid” electrode arrays in cats. Hybrid electrode arrays (Figure 1A) consisting of eight 1 mm long Blackrock electrodes (Blackrock Microsystems, Salt Lake City, UT, United States), four 1.5 mm long planar silicon probes (NeuroNexus, Ann Arbor, MI, United States), and four “short” 1 mm long and four “long” 2 mm long microwires (Microprobes, Gaithersburg, MD, United States) were implanted in the post-cruciate gyri of male cats. All electrodes had an active site at 1 mm depth. The implantation duration for the cat whose histological data was analyzed in this study was 371 days, after which the cat was fully anesthetized and transcardially perfused as described previously (Han et al., 2012). After post-fixation, the implanted device was carefully removed (Figure 1B), and a tissue block 6 mm × 8 mm × 10 mm was punched out. Horizontal serial sections 50 μm thick were sectioned perpendicular to the electrode shanks to a depth of 3 mm below the brain surface, encompassing the full length of all the electrode shanks, for a total of 60 sections. The tissue sections were stained using immunofluorescence labeling procedures as described previously (Duong and Han, 2013). Briefly, free-floating sections were subjected to 24 h high-intensity LED photoirradiation in citrate buffer to reduce autofluorescence, followed by 1 h antigen retrieval at 83°C, cooling to room temperature, 2 h blocking with 5% normal goat serum, 48 h incubation at 4°C with constant agitation with primary antibodies, then the same incubation for secondary antibodies (Alexa Fluor, Life Technologies, Carlsbad, CA, United States). All tissue sections were multiple-labeled with rabbit anti-IBA-1 (Wako Chemical, Richmond, VA, United States), biotinylated mouse anti-NeuN (EMD Millipore, Billerica, MA, United States), chicken anti-MAP-2 (EMD Millipore, Billerica, MA, United States), rat anti-GFAP (Life Technologies, Carlsbad, CA, United States), and DRAQ-5 nuclear stain (eBioscience, San Diego, CA, United States). IBA-1, MAP-2, GFAP, and DRAQ-5 were used for alignment purposes only. Only NeuN was quantified in this study. Sections were mounted in mounting media on glass slides with 50-μm-thick square-shaped polyimide spacers cut from adhesive film (Product No. 2271K69, McMaster Carr, Los Angeles, CA, United States) placed between the slide and the coverslip to avoid compressing the sections. The slides were imaged using an LSM 510 Meta laser scanning system attached to a Zeiss Axiovert 200 M inverted microscope with an Olympus 20× objective (N.A. = 0.5). A 10 × 10 grid of images spanning about 3.5 mm × 3.5 mm with 10% overlap was obtained at each *z*-depth or “optical slice.” All electrode tracks were included in the scanned area. Optical slices were collected from slightly above the top to slightly below the bottom of the section in order to capture all of the tissue. Constant laser power was used. The voxel dimension was 0.89 μm × 0.89 μm × 3 μm. The scan time was approximately 5 h per section. All of the above was carried out at our group’s previous institution, Huntington Medical Research Institutes (HMRI), in Pasadena, CA, United States. All procedures involving animals were approved by the HMRI Institutional Animal Care and Use Committee.

### Image Processing and 3D Reconstruction

#### Overview

**FIGURE 2:**
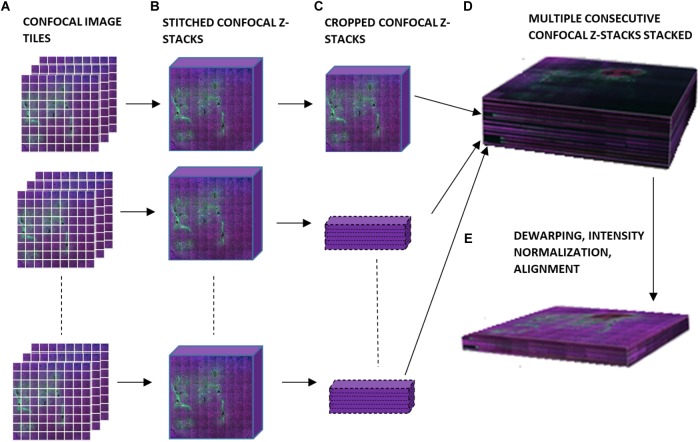
Schematic of workflow for 3D image reconstruction, illustrating **(A)** confocal image tiles, **(B)** stitched confocal *z*-stacks, **(C)** confocal *z*-stacks cropped to the same *x* and *y* dimensions, **(D)** stacked confocal z-stacks, and **(E)** the final 3D volume after dewarping, intensity normalization, and alignment.

Figure 2 is a schematic of the steps involved in the 3D reconstruction process. The confocally-scanned images (Figure 2A) were first stitched. This created a 3D stack 50 μm thick for each section (Figure 2B). Then, all of these were cropped to the same *x-y* size (Figure 2C) and stacked consecutively to form one 3D volume (Figure 2D). The stacks were not aligned at this point, but putting them in one file streamlined the subsequent steps. Dewarping, intensity normalization, and alignment were then performed to reconstruct the volume of tissue (Figure 2E). Finally, electrode tips were drawn, neurons were identified, and quantifications of neuronal density at various distances from the electrode tip were obtained. All these steps are described in detail in the succeeding sections. The computer used was a PC running 64-bit Windows 10 with 176 GB RAM, two quad-core Intel Xeon 2.13 GHz processors, and an ATI FirePro V7800 graphics card.

#### Stitching, Cropping, and Stacking

**FIGURE 3:**
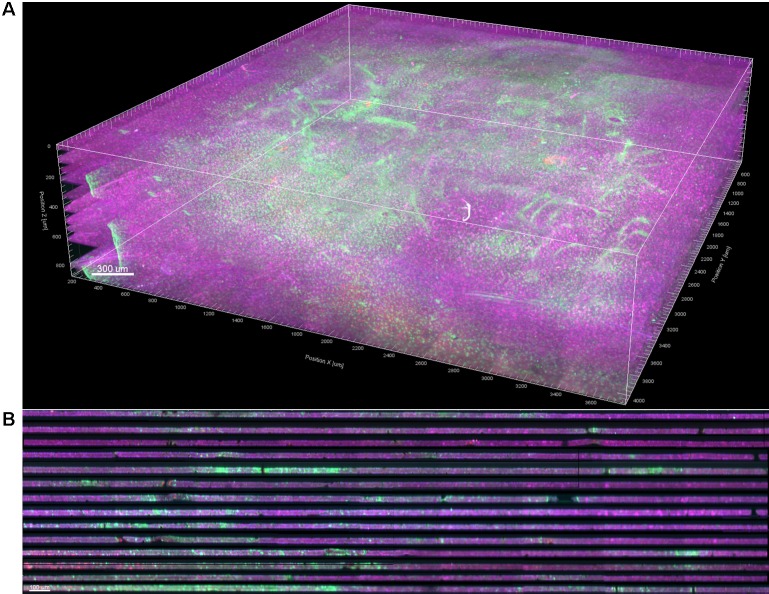
**(A)** 3D volume rendering of GFAP (green) and NeuN (magenta) after stitching, cropping, and stacking. Dewarping, alignment, and intensity normalization have not yet been performed. **(B)** Vertical cross section through the 3D volume. The dark bands are dark, out-of-focus optical slices above and below the actual tissue in each section.

Stitching was performed in XuvStitch (Emmenlauer et al., 2009) (Figure 2A,B). Individual image tiles were stitched using automatic mode with ten percent overlap and ninety-five percent similarity threshold. Manual stitching was seldomly used only when automatic mode was not adequate. The *x-y* size of the largest individual section for stacking was 4553 × 4658 pixels. Differences in the *x* and *y* dimensions of each section were eliminated by cropping them to equal *x* and *y* values, i.e., 4,093 × 4,184 (Figure 2C) before stacking them in one 30-GB file (Figure 2D). For the dataset shown in this paper, fourteen consecutive physical sections were stacked, because they had all electrode tips and easily-identifiable tracks that could be used later on for alignment. Figure 3A shows the image data after cropping and stacking. At this point, the physical sections are totally un-aligned.

#### Dewarping and Intensity Normalization

Dark bands between sections in the stack were observed (Figure 3B). Most of this was from imaging extra optical slices above and below the tissue to ensure that no data was missed, and could be corrected by simply deleting the extra slices. However, many sections were tilted relative to the imaging plane or had significant curvature or wrinkles from mounting, so deleting slices would have resulted in either losing data or including dark areas. To remedy this problem, we developed a custom dewarping program (Supplementary File 1) in MATLAB (The MathWorks, Inc., Natick, MA, United States) that interfaced with Imaris (Bitplane AG, Zurich, Switzerland) via the ImarisXT feature. ImarisXT causes MATLAB m-files stored in specific folders to appear as available actions in the Imaris menus. Clicking on one of these actions starts MATLAB and executes the m-file. A Java library provided by Bitplane provides extra functions that can be written into the MATLAB code to perform various operations such as transferring data between the two programs.

When executing the dewarping program, the user is first prompted to input the number of optical slices per section the user wants to keep. We chose sixteen slices per section because the tissue block was sectioned at 50 μm thickness. Each optical slice is 3 μm thick, hence 16 μm × 3 μm = 48 μm (approximately 50 μm).

**FIGURE 4:**
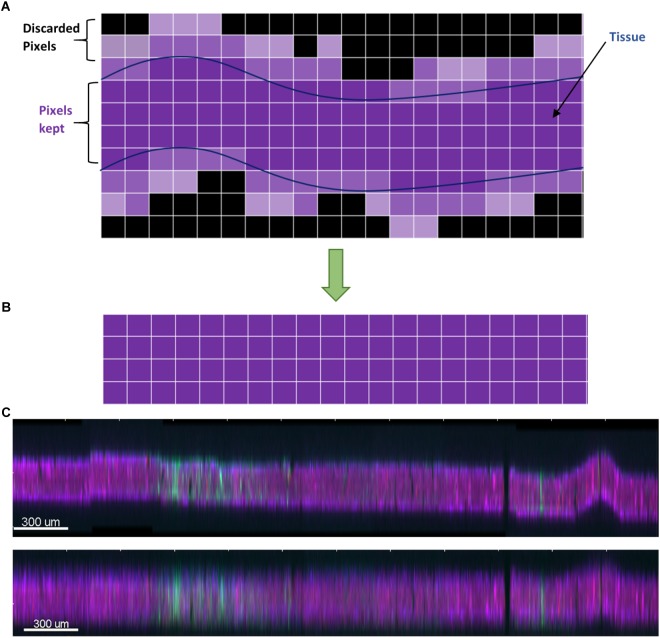
**(A)** Schematic of the dewarping algorithm. The brightest contiguous subset of voxels in *z* is retained while the rest are discarded, resulting in a flatter section. **(B)** Vertical cross section through a section before dewarping with height scaled 10×. The section is tilted, has a large wrinkle, and has a stitching artifact. **(C)** After dewarping the tissue is nearly flat (height still scaled 10×).

The algorithm then chooses the brightest contiguous subset of voxels in each *z*-column and retains them, while discarding the low intensity voxels at the top or bottom (Figure 4). The dark slices with no useful information encompassing dark, out-of-focus voxels are removed, and because each column of voxels is dealt with separately, any tissue tilt, curvature, or wrinkling is also fixed. The *x-y* data are not modified, so there is neither addition nor removal of horizontal distortions, and no alignment is performed at this stage. An additional step of intensity normalization using an in-built function in Imaris was also applied to correct differences in staining intensity from section to section, assisting in qualitative analysis and improving segmentation of nuclei. Intensity-based quantification was not performed in this study, but if it was, the intensity normalization step would have been skipped to avoid introducing systematic error.

#### Alignment

**FIGURE 5:**
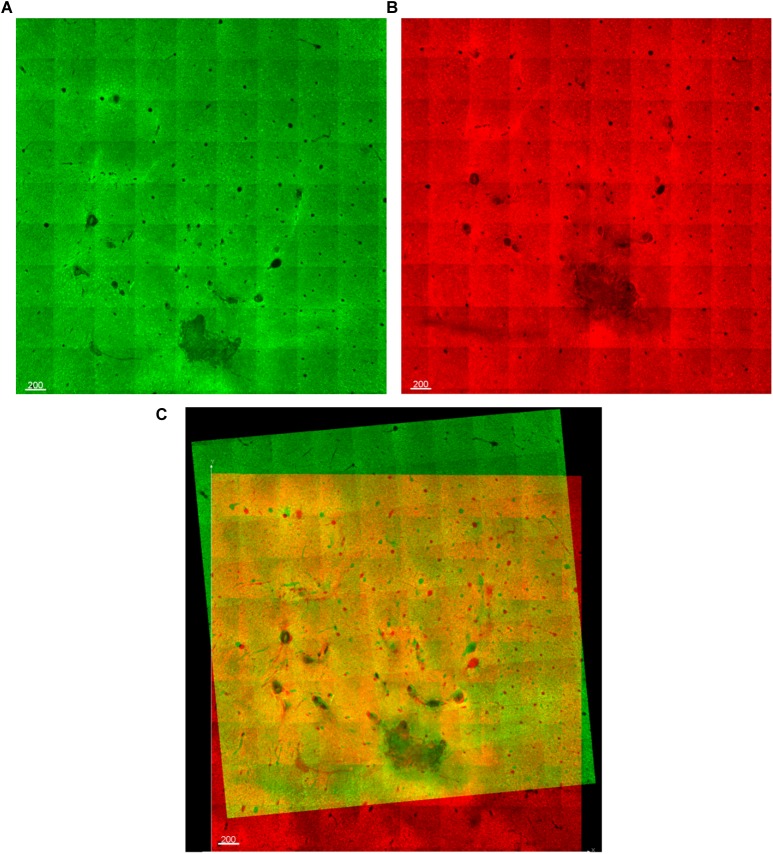
**(A,B)** Two optical slices from vertically-consecutive sections. One is shown in green, the next in red. **(C)** During alignment, the red and green images are overlaid, allowing fiducials (electrode tracks) to be manually aligned.

Next, manual alignment of the stacked, dewarped image dataset was done using AutoAligner (Bitplane AG, Zurich, Switzerland). This software has both automatic and manual alignment mode. However, automatic alignment works best for image data having very large and obvious fiducials, such as the outer boundary of the tissue sample, which we did not have in our dataset. Instead, we applied manual translational and rotational transformations by using the Microprobes and Blackrock electrode tracks that we intended to perform quantification and analysis on as fiducials. The electrode tracks were identified as holes devoid of staining and with characteristic size and arrangement. Figure 5 shows the alignment process, which involved overlaying the bottom optical slice of one section with the top optical slice of the next vertically-consecutive section in two different colors. By aligning adjacent optical slices, rather than whole sections, the impact of angled or curved fiducials is greatly reduced: horizontal displacement of an angled fiducial will still occur throughout the 16 optical slices of the section, even if it does not occur between the optical slices that are aligned. AutoAligner’s manual alignment interface was very useful, but the program crashed when attempting to apply the planned translations and rotations to datasets larger than about 12 GB. Therefore, we created a custom software program in MATLAB that would take saved alignment data (.aln files) from AutoAligner and apply the specified translations and rotations to a dataset in Imaris, using the ImarisXT feature to allow communication between MATLAB and Imaris (Supplementary File 2). The program accomplishes the same rotations and translations as AutoAligner, but does not run into file size problems. Briefly, the .aln file must be renamed with the .txt extension, then MATLAB opens and reads the text file, calculates the rotations and translations to be performed in MATLAB (which uses different conventions), orders Imaris to pad (add black space) the margins of its dataset to the exact minimum extent necessary, then loads, rotates, translates, and sends back each optical slice one at a time to Imaris. Sending one slice at a time is necessary because while Imaris and MATLAB have extremely large memory limits, the Java interface handling data transfer does not.

#### Drawing Recording Sites and Quantification

**FIGURE 6:**
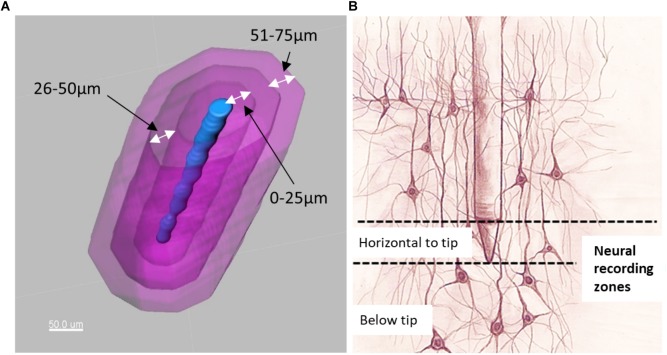
**(A)** Electrode tip (blue) and distance bins (magenta) for quantification of neurons. **(B)** Illustration of neurons located horizontal to the electrode tip versus those located below the electrode tip.

The data set was first converted from 16-bit to 32-bit and down-sampled by fifty percent before drawing the shanks. This is a requirement for the distance transform in Imaris. Artificial recording sites were manually drawn using the “surfaces” feature in Imaris (Figure 6). Electrode tip lengths, i.e., lengths of the active electrode sites, for the Blackrock electrodes and the 1-mm microwire electrodes were previously measured under an optical microscope during device assembly. Using this information, the section where the recording site begun was determined by measuring the distance backward from the deepest point of the electrode track. The “circle” drawing mode was used to draw the recording sites and 100 vertices were chosen to provide smoother zones. Once the contours were drawn on sequential slices, Imaris built the recording site by connecting the contours. Care was taken to ensure that adequate contours were drawn to allow the formation of a smooth surface.

Distance transformations were applied to each shank in Imaris. This computes the distance from each voxel in the image to the nearest point on the shank surface. Then, surfaces were created around the shank to define distance bins in increments of 25 μm up to a distance of 200 μm (Figure 6A). These distance bins could be further subdivided by depth, as shown schematically in Figure 6B.

**FIGURE 7:**
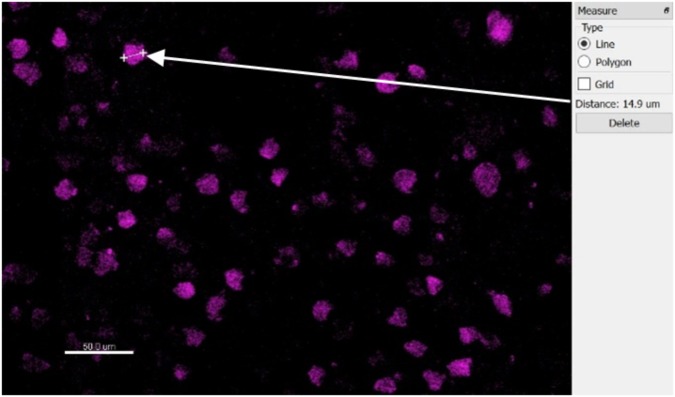
Top-down slice view of NeuN-stained tissue showing one measurement of a typical neuron’s diameter.

**FIGURE 8:**
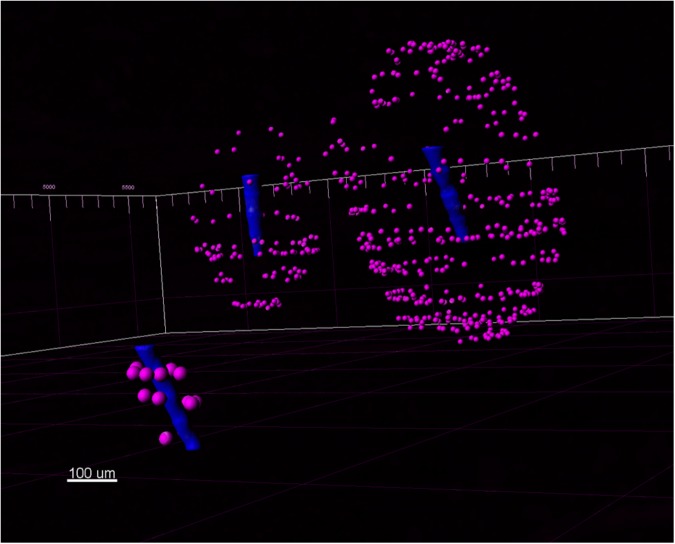
A view of three Blackrock shank tips (blue) with neurons (magenta) detected in the 3D reconstructed volume.

The automatic spot detection feature of Imaris was used to identify neurons. Neuronal nuclei are distinguishable as high-intensity spheroids in the NeuN channel having a diameter of about 10 μm. This diameter was obtained by measuring multiple NeuN spheroids in 2D slice views (Figure 7). The size requirement also helps avoid double-counting of the same neuron in two vertically-adjacent sections when it is cut in half by the vibratome blade. Intensity mean thresholds were applied to include spots only in a certain range of intensity values. False negatives were manually removed after automatic detection. Neurons in various distance bins surrounding three Blackrock electrode tips are shown in 3D space in Figure 8. Neuron counts were then converted into neuronal density by dividing by the volume of the bin.

## Results

### Qualitative Analysis of 3D Reconstructed Tissue Volume

**FIGURE 9:**
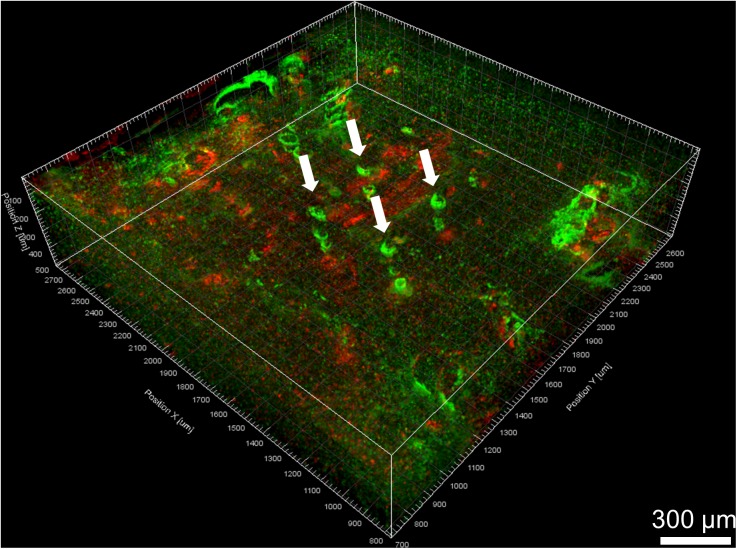
Volume rendering of GFAP (green) and IBA-1 (red) after alignment. Four Blackrock electrode tracks are highlighted with white arrows. The astroglial sheath stained brighter in some sections than others and looks like a series of rings.

**FIGURE 10:**
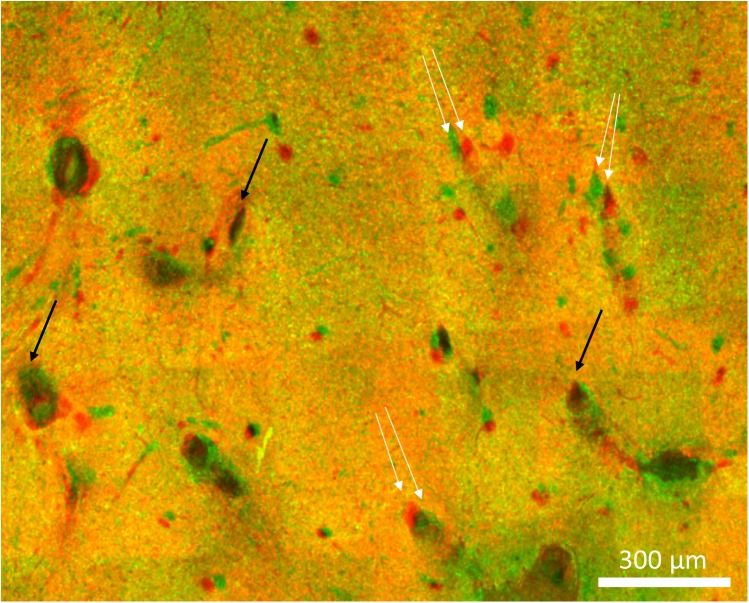
Imperfect alignment of two sections. Black arrows indicate well-aligned fiducials whereas white arrows indicate fiducials that could not be aligned presumably due to distortions in the tissue. The magnitude of the misalignments indicated are 30, 42, and 38 μm.

A 3D view of the reconstructed tissue volume is shown in Figure 9. Several electrode tracks appear as a series of concentric rings (due to inconsistent staining intensity, even after intensity normalization). The quality of alignment was sufficient for large-scale overall viewing as well as close-up viewing and quantification near the electrode tracks. The alignment was within about 40 μm, as shown in Figure 10. In order to achieve perfect alignment, local stretching of the image will be necessary to undo the non-uniform stretching, compression, and angling of tissue during immunohistochemistry and mounting.

The 3D reconstruction appeared relatively seamless when viewed from the side except for thin dark bands between sections. It is likely that the thickness of each section during imaging was less than the thickness after sectioning due to shrinkage and/or compression of the section during immunohistochemistry and mounting. In the future this could be corrected by keeping fewer optical slices during dewarping and then scaling the image up to its actual dimensions. This artifact would not affect quantification, however, because the same numbers of neurons are present in each section.

A significant tilt of the electrodes was observed. Since the tissue punch was inserted perpendicular to the brain surface and the block of tissue was then sectioned horizontally, this observed tilt represents the actual angle of the device in tissue. The device was observed to be tilted and sunken down into cortex at necropsy. Tethering forces and the build-up of fibrotic tissue have been reported to tilt electrodes over time (Barrese et al., 2013; Nolta et al., 2015; Cody et al., 2018). Being able to observe this tilt so clearly is a significant advantage of 3D vs. 2D analysis. It was also noted for this explanted device that the Blackrock and short microwire electrode shanks were still straight while the NeuroNexus and long microwire electrodes had been bent to an angle different from their original position (Figure 1B).

The superficial cortex near the base of the array was not included in the reconstruction because the electrode tracks were not visible in this area. As can be seen in Figure 1B, fibrotic tissue had encapsulated the base of the array and remained adhered to it during explantation, similar to other reports using Blackrock arrays (Barrese et al., 2013; Cody et al., 2018). However, very little, if any, tissue adhered to most of the length of the shanks, and there were no missing chunks of tissue in the deeper histological sections. Also, the brain did not appear to be torn or stretched as a result of removing the array.

To evaluate the consistency of manual alignment across users, the alignment rotations and translations of two users were compared. When each user performed the alignment according to their own judgment, the average absolute difference was 4.4 ± 4.6° rotation, 170 ± 81 μm translation in *x*, and 102 ± 44 μm in *y*. When each user was instructed to use the same set of features as fiducials, the average absolute difference was 0.4 ± 0.6° rotation, 9 ± 16 μm translation in *x*, and 13 ± 21 μm in *y*.

### Quantitative Analysis of Neuronal Density Near Recording Sites

**FIGURE 11:**
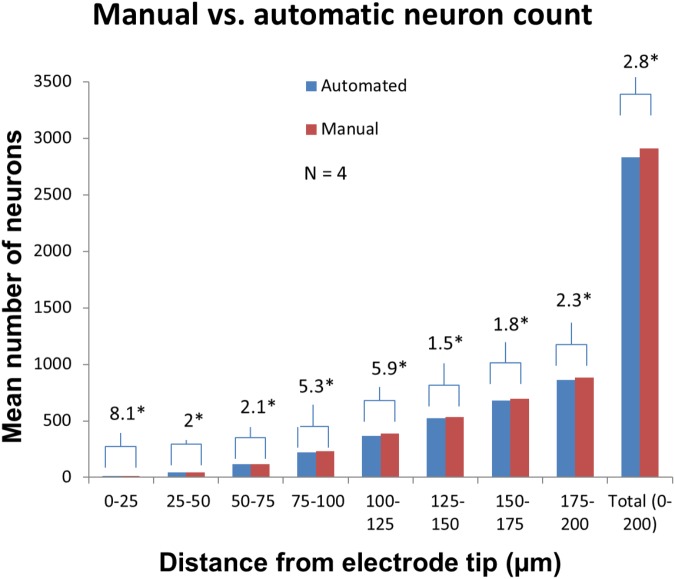
Manual validation of automatic neuron counting protocol. From four electrodes analyzed, on average, manual counting labeled 2.8% more neurons than automatic counting. The greatest errors were seen in the 0–25, 75–100, and 100–125 μm bins. Asterisk denotes statistical significance (*p* < 0.05) using one-tailed paired *t*-tests.

Manually validating the accuracy of our neuron counting technique for *N* = 4 electrodes showed that automatic neuron counting slightly underestimates the number of neurons in the first 200 μm near an electrode by an average of 2.8%. Figure 11 shows the numbers counted in each bin manually vs. automatically. As another check on quantification accuracy, and to provide a reference point for neuron quantification near electrode tips, we automatically counted neurons in large regions of healthy neural tissue at the same depth as the electrode tips but far away horizontally. By this method we calculated a density of 48,000 neurons/mm^3^ in healthy tissue.

**FIGURE 12:**
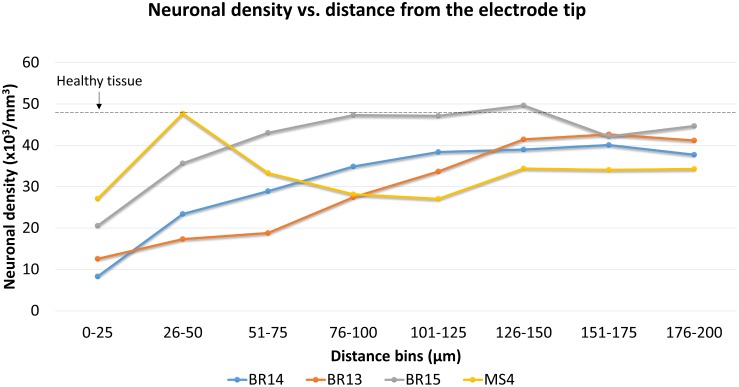
Neuronal density for three Blackrock electrode tips and one short microwire electrode tip. For Blackrock electrodes (BR), neuronal densities increased with distance from the electrode surface. For the microwire electrode (MS), neuronal density was higher than the Blackrock electrodes in the first 50 μm, then decreased until it was less than the Blackrock electrodes from 100–200 μm. Dashed line represents the neuronal density measured in healthy tissue far from the electrode tracks at the same depth.

Neuronal density was then quantified for three Blackrock electrodes and one short microwire electrode as a proof-of-concept (Figure 12). No statistical inferences were attempted due to the small *N*. For the Blackrock electrodes, neuronal density was lowest 0–25 μm from the electrode surface, increased by more than two-fold by 76–100 μm, then generally leveled off to a constant value somewhat below healthy tissue by 126–150 μm. Substantial variability was present even in this small sample. Meanwhile, the short microwire electrode had a higher density than the Blackrock electrodes in the first 50 μm, then decreased until it was less than the Blackrock electrodes from 100–200 μm. Future investigations of more microwires will be necessary to elucidate whether this pattern is typical of microwires or was an outlier. Actual neuron counts for the Blackrock electrodes were approximately 10 in the 0–25 μm bin, 200 total in the four bins from 0–100 μm, and 1000 total in the four bins from 100–200 μm.

**FIGURE 13:**
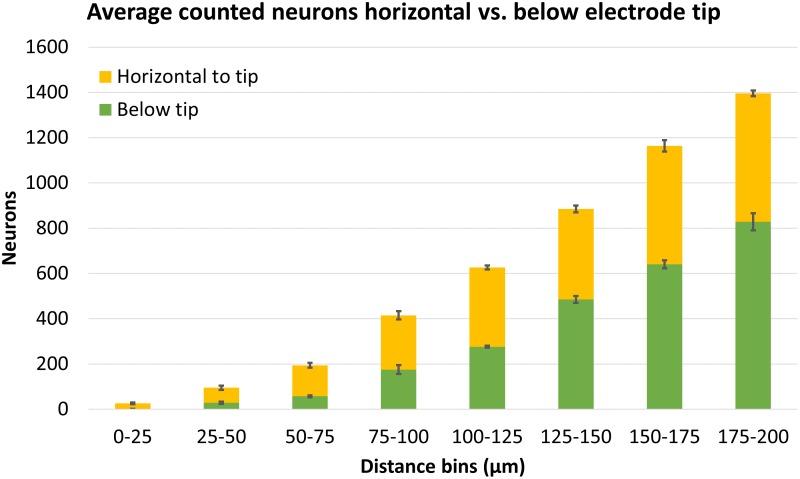
Average neuron counts surrounding four Blackrock electrodes, split in two different depths: horizontal to the electrode tip, or below the electrode tip. Error bars show standard deviation.

Finally, we compared counts of neurons found in areas horizontal to the electrode tip (which could have been counted using traditional 2D analysis) versus counts in areas below the electrode tip (which can only be counted using 3D analysis). These regions are shown schematically in Figure 6B. Figure 13 shows the average number of neurons counted around four Blackrock electrode tips in the tissue horizontal to vs. below them. A substantial portion of neurons are found below the electrode tips, especially at farther distances. Combining counts for 0–100 μm, 36% of neurons were found below the tips, and for the whole 0–200 μm, 52% were below the tips.

### Total Time Required

For an experienced user performing this analysis on a typical-sized stack (∼15 sections), the process required 2.5 h for stitching, 1 h for cropping, 8 h unattended process time for dewarping, 1.5 h for manual alignment, and 12 h unattended process time to apply the alignment, for a total of 5 h labor plus two overnight steps. For the quantification steps, each electrode tip required 0.5 h to draw the tip, 4 h to create distance bins (mostly unattended process time), and 2.5 h to detect and quantify neurons, for a total of 3 h labor plus 4 h mostly unattended process time. Scaled up to an array of 20 electrodes, the entire process would take 65 h of labor, 80 h mostly unattended process time, and 20 h completely unattended process time.

## Discussion

Confocal images of 50 μm serial sections of cat cortex were successfully dewarped, aligned, reconstructed in 3D, and quantified in terms of neuronal density at various distances from electrode tips. To our knowledge, this is the first time that serial sections have been reconstructed around an implanted electrode array. This technique has the advantage of providing a holistic view of the entire implant area and allowing for quantification in more accurate, 3D representations of electrode recording zones. Specifically, electrode tilt was easily visualized (which is not the case in 2D histology) and neurons were quantified below the tips of the electrodes (which is not possible in 2D histology). Within the first 200 μm, 52% of neurons were found below the tips; in 2D histology, these neurons would have been left out. This may have ramifications in correlation of recording quality with tissue response metrics, especially since neurons below the tip are presumably healthier. Our dewarping algorithm and pipeline for manual alignment is not specific for neural electrodes and could also readily be applied to other datasets which require relatively precise alignment of fiducials that are difficult or impossible for software to align automatically.

Our measurement of 48,000 neurons/mm^3^ in healthy tissue is plausible. This value is only slightly higher than the average density in the farthest distance bin (175–200 μm) of 39,000 neurons/mm^3^, indicating that there may be some loss of neurons 200 μm from the electrode shanks. Alternatively, the brain under the array may have been compressed such that the tissue horizontal to the tips was actually a different cortical layer. Neuronal density varies by brain region, cortical depth, and animal species. From an earlier study, averaging the neuronal density of 72 non-stimulating electrode tips in nine cats in the farthest bin (120–150 μm) in the same region of cat cortex (post-cruciate gyrus), one obtains a neuronal density of approximately 570 neurons/mm^2^, which can be divided by the thickness (three 7-μm sections) to get an estimate of 27,000 neurons/mm^3^ (McCreery et al., 2010). That number is roughly similar but substantially smaller than our measurement, even when compared to the average density we measured in the 125–150 μm in our study, 41,000 neurons/mm^3^. Differences in depth (1 mm vs. 1.1–1.2 mm), differences in histological method (free-floating vs. paraffin), or large-scale effects of the devices (hybrid vs. microwire arrays) may explain this discrepancy.

An alternative to using our technique is tissue clearing. Tissue clearing allows staining and imaging of very thick (greater than 1 mm) samples, eliminating the need for digital reconstruction, and eliminating the risk of mishandling/losing one section and having to interpolate its alignment and quantification. However, tissue clearing techniques require specialized equipment (Chung et al., 2013), multi-week processing (Yang et al., 2014), or outsourcing to private companies. Special long-working-distance objectives are also required. In addition, although digital reconstruction introduces minor distortions, clearing can cause major expansion, contraction, reductions in fluorescent signal, or changes in ultrastructure that need to be characterized and troubleshot (Richardson and Lichtman, 2015; Azaripour et al., 2016). This is especially problematic for quantification, where accurate absolute dimensions and consistent staining are important. The presence of electrode tracks, voids, dense fibrotic tissue, or a device left in place may create further complications in achieving uniform dimensions, clearing, and staining. Therefore, for labs that have not yet mastered tissue clearing, the ability to produce 3D reconstructed datasets using standard immunohistochemical methods is still appealing.

There are many areas for improvement in our technique. Neurons near the NeuroNexus probes were not quantified in this study. To do so, it will be necessary to measure backward from the tip of the shank to the known locations of the recording sites. The dewarping algorithm could be improved by using a physical model of the section as an elastic material, so that it is stretched realistically in *x* and *y* whenever *z* is adjusted. Physical models such as thin-plate splines have been used in automatic reconstruction strategies (Anderson et al., 2009; Wang et al., 2014). Even if this is done, distortions may persist due to anisotropic shrinkage or expansion of tissue during immunohistochemistry and mounting. In this case, it may be necessary to allow local stretching and compression during alignment as well. Addressing these distortions would be likely to improve alignment consistency – since the distortions make it impossible to align all fiducials simultaneously, the user has to decide which fiducials to prioritize, leading to high variability in alignment unless the same fiducials are used. Our method could also benefit from making the alignment and tip-drawing steps at least semi-automated to improve consistency and save time. Optimizing the software could also save time. Profiling the execution time of the MATLAB code revealed that the ImarisXT functions passing data between Imaris and MATLAB account for the majority of the code’s execution time and cause large files to take several hours to process. A software solution that does not rely on ImarisXT could be much faster. Finally, it is difficult to understand what is happening in complex, multi-channel, 3D histological datasets when they are simply volume rendered and displayed on a flat screen. It may be worthwhile to explore derived data such as intensity averages or use tools to explore the data in virtual reality.

In the future, we intend to analyze the full cohort of cats implanted with hybrid electrode arrays. This cohort has chronic neural recording data and explanted device SEM images, so the correlations and connections between these data sets are likely to provide new insights into chronic failure modes and how they differ among different types of electrodes.

## Ethics Statement

The animal studies were approved by the Animal Care and Use Committee of HMRI and were performed under the guidelines set forth in the Guide to Care and Use of Laboratory Animals.

## Author Contributions

AN developed the software techniques and processed all data. NN developed the dewarping program and alignment workaround program. MH designed the device, conducted the animal studies, and supervised the study. All the authors wrote the manuscript.

## Conflict of Interest Statement

The authors declare that the research was conducted in the absence of any commercial or financial relationships that could be construed as a potential conflict of interest.
